# Virulence Factors in Coagulase-Negative Staphylococci

**DOI:** 10.3390/pathogens10020170

**Published:** 2021-02-04

**Authors:** Angela França, Vânia Gaio, Nathalie Lopes, Luís D. R. Melo

**Affiliations:** Laboratory of Research in Biofilms Rosário Oliveira, Centre of Biological Engineering, University of Minho, 4710-057 Braga, Portugal; vaniagaio@ceb.uminho.pt (V.G.); nathalie.lopes@ceb.uminho.pt (N.L.)

**Keywords:** coagulase-negative staphylococci, biofilms, virulence factors

## Abstract

Coagulase-negative staphylococci (CoNS) have emerged as major pathogens in healthcare-associated facilities, being *S. epidermidis*, *S. haemolyticus* and, more recently, *S. lugdunensis*, the most clinically relevant species. Despite being less virulent than the well-studied pathogen *S. aureus*, the number of CoNS strains sequenced is constantly increasing and, with that, the number of virulence factors identified in those strains. In this regard, biofilm formation is considered the most important. Besides virulence factors, the presence of several antibiotic-resistance genes identified in CoNS is worrisome and makes treatment very challenging. In this review, we analyzed the different aspects involved in CoNS virulence and their impact on health and food.

## 1. Introduction

Staphylococci are a widespread group of bacteria that belong to human and animals normal microflora [1]. *Staphylococcus* genus comprises two main groups, the coagulase-negative staphylococci (CoNS) and coagulase-positive staphylococci (CoPS), which were defined according to their ability to produce the enzyme coagulase [2]. *Staphylococcus* species were first characterized by Friedrich Rosenbach, who established that yellow/orange colonies corresponded to CoPS species and white colonies to CoNS [3]. Among Staphylococci, *Staphylococcus aureus*, belonging to the CoPS group, and *Staphylococcus epidermidis*, from the CoNS group, are the most frequently isolated of each group, the reason why most of the CoNS studies are focused on these species [4]. Nonetheless, CoNS cover a large and continuously expanding group of bacteria, with more than 50 species described so far, which are currently distributed into 41 main species, divided into more than 20 subspecies (reviewed in [5]). Since CoNS are common colonizers of human skin, they have been recurrently considered culture contaminants rather than recognized as the causative agent of important infections [6,7]. Despite their benign interaction with the host, it is now known that these species can cause critical infections, especially in immunocompromised patients, the reason why they are currently acknowledged as opportunistic pathogens and have been gaining increasing importance in the healthcare field (reviewed in [8,9]).

The increase of CoNS impact on the clinical field was emphasized by the extensive medical progress, where the use of implantable medical devices and the increasing number of vulnerable patients have allowed CoNS to cause significant infections in humans [10]. More importantly, these factors have elevated the number of morbid, chronically ill and immunocompromised patients, as well as the mortality rates related to CoNS [11,12]. Moreover, as CoNS are known to colonize both farm and domestic animals, they may as well establish infections upon opportunity, although to a lesser extent. For instance, several bovine mastitis infections associated with CoNS have increasingly being reported over the years [13,14,15]. Additionally, several studies demonstrated that infections caused by these species affect debilitated domestic animals as cats with conjunctivitis, upper respiratory tract and skin or wound infections [16], or dogs with keratitis or urinary tract infections [17,18]. This is especially concerning, since it was already demonstrated that CoNS may be transferred from pets to their owners [19,20]. Moreover, some farm animals such as chickens are known to be the main reservoirs of antimicrobial resistance genes [21]. Importantly, CoNS have also been detected as contaminants of food products. Contamination with CoNS has been found in ready-to-eat foods of animal origin [22,23], retailing raw chicken meat [24], and in bulk tank milk or minced meat [25]. Altogether, the infections caused by CoNS species have become more frequent and harmful to both humans and animals, and, subsequently, entail an increase in the economic burden [26].

The virulence factors of CoNS have been considered to a lesser extent than CoPS (e.g., *S. aureus*) since they are coagulase “free”. Nevertheless, the continuous findings and updates on species and subspecies have revealed a heterogeneous group, ranging from nonpathogenic to facultative pathogenic species, with distinct virulence potential levels [27]. Some isolates have become increasingly concerning, as *Staphylococcus lugdunensis*, which lately has been recognized as a pathogenic bacterium with a high virulence impact [28]. *S. lugdunensis* can cause highly acute and destructive events of infective endocarditis (IE), leading to higher mortality rates than other CoNS species, which generally cause less severe infections [29]. Despite some similarities with *S. aureus*, CoNS are generally less pathogenic and present a smaller array of virulence factors, being less studied than the major CoPS pathogen (reviewed in [8]). Nevertheless, these species deserve special attention due to their significant impact on the clinical and food fields, resulting from several virulence factors. Colonization of surfaces and formation of biofilms by CoNS bacteria has long been considered their main virulence factor, being known that the heterogeneity of bacteria within biofilms may contribute to their persistence with emphasis on persister cells, viable but non-culturable (VBNC) cells and small colony variants (SCVs). Moreover, resistance to antibiotics and the production of bacteriocins and enterotoxins are aspects also contributing to their virulence.

In order to enhance the knowledge on CoNS pathogenicity, these virulence factors and their impact on health and food will be further discussed in this review.

## 2. Adhesion and Biofilm Formation

**Figure 1:**
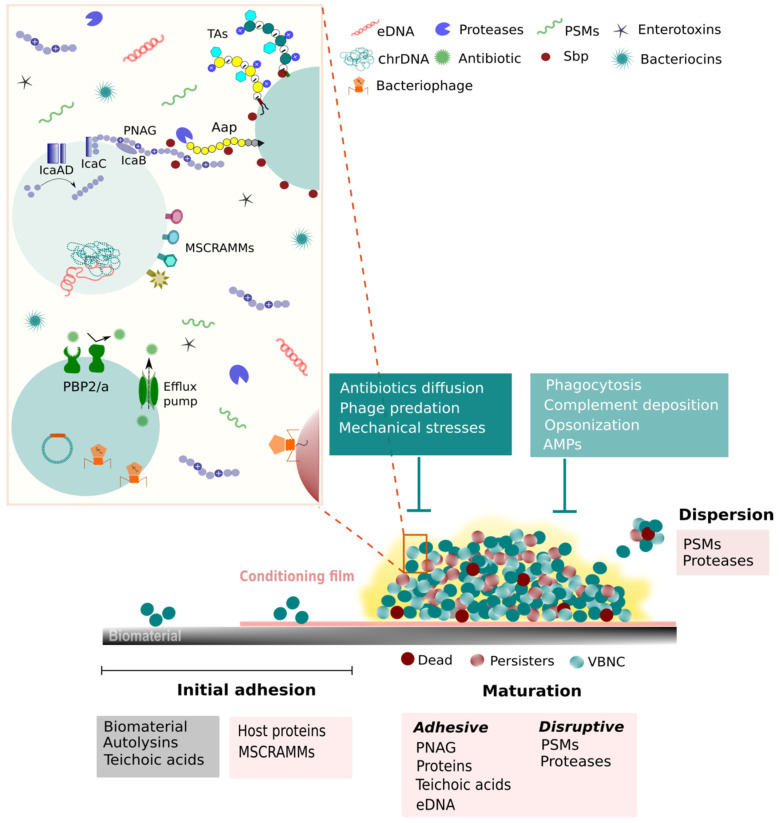
CoNS virulence factors summary illustration. CoNS species are equipped with several strategies to overcome less favorable conditions and, thus, to survive in a variety of different environments. Amongst all strategies, the capacity to form biofilms is one of the most important. Biofilm formation starts with the adhesion of free-floating cells to a surface, either abiotic or biotic, and proceeds through the division and aggregation of cells, which creates the characteristic multi-layered structure. In addition, an extra-polymeric protective matrix is produced by the cells. This is defined as the maturation phase. This phase is mediated by adhesins, but also by molecules with disruptive properties, such as PSMs, since these are necessary to form channels that ensure the flow of nutrients to all biofilm layers. Moreover, as could be expected, PSMs have a pivotal role in the final step of the biofilm lifecycle, the dispersion, as it allows biofilm cells to escape and colonize other places. It is important to stress that, in the illustration, only a brief description of some of the molecules involved in the several mechanisms employed by CoNS to respond and subsist to external stresses are depicted. Aap, accumulation associated protein; AMPs, Antimicrobial peptides; chrDNA, chromosomal DNA; eDNA, extracellular DNA; MSCRAMMs, Microbial surface components recognizing adhesion matrix molecules; PBP2/a, penicillin-binding protein 2 and 2a; PNAG, poly-N-acetylglucosamine; PSMs, phenol-soluble modulins; Sbp, Small basic protein; TAs, teichoic acids; VBNC, Viable but-non culturable cells.

In the natural, industrial, and clinical environments, bacteria grow predominantly in biofilms. Biofilms are multicellular and structured communities of microorganisms adhered to a substratum and embedded in a matrix of extracellular polymeric substances [30]. These communities provide protection from several external stresses such as antimicrobial agents [31] and the attacks mounted by the host defenses [32,33] facilitating, thus, the survival of the cells inside the biofilm. As referred to above, CoNS capacity to form biofilms is considered a major virulence factor and, thus, the mechanisms underlying biofilm formation gained special attention in the last decades. Biofilm formation by CoNS is an intricate and multistep process that can be primarily divided into three phases: (i) adhesion or attachment to a surface, (ii) maturation into a complex multicellular structure and (iii) dispersion of cells into the surrounding environment [34] (Figure 1).

Surface-associated adhesins have an important role in biofilm formation, both in the initial adhesion to host proteins and tissues, and biofilm maturation. These adhesins comprise covalently and non-covalently anchored proteins, as well as non-proteinaceous factors (reviewed in [35,36,37,38]). Covalently anchored proteins, or also called cell wall-anchored (CWA) proteins, are characterized by the presence of the LPXTG motif, which is recognized by the sortase enzyme that, in turn, conducts the process of anchoring the protein to the peptidoglycan [39]. Briefly, in *S. epidermidis*, CWA proteins can be divided, based on the presence of common characteristic domains, into two main families [36]: (i) microbial surface component recognizing adhesive matrix molecules (MSCRAMM), which integrate members of the serine-aspartate repeat (Sdr) and *S. epidermidis* surface (Ses) proteins and (ii) G5-E repeat proteins family that includes the accumulation associated protein (Aap). A defining feature of the MSCRAMMs family is the presence of two tandemly linked IgG-like folded domains, which can engage in ligand binding by the “dock, lock and latch” mechanism [40]. This mechanism enables a stable adhesin-ligand complex which is important to ensure a tight binding under the fluid shear forces that frequently occurs around indwelling medical devices (IMD) [40]. Moreover, there are also uncategorized CWA proteins putatively involved in biofilm formation as, for instance, biofilm-homolog protein (Bhp) [36]. Within the group of the non-covalently anchored cell wall are the autolysins/adhesins AtlE and Aae. Lastly, the non-proteinaceous group is composed of teichoic acids (TAs) and the polysaccharide intercellular adhesion (PIA) [35,36,37,38]. The function of these molecules will be briefly discussed in this section in the context of their contribution to biofilm formation.

### 2.1. Initial Adhesion

CoNS have the capacity to adhere to several different surfaces including abiotic (polyethylene, stain steel, rubber, and glass) or biotic surfaces (living tissue or abiotic surfaces covered with proteins), the former being more relevant in the context of food processing industry [41] and the last more relevant in clinical settings [42].

#### 2.1.1. Initial Adhesion to Abiotic Surfaces

The adhesion to abiotic surfaces is primarily mediated by non-specific physicochemical forces such as hydrophobic and electrostatic interactions [43,44]. Nevertheless, specific bacterial surface molecules can also foster this process.

AtlE, a major autolysin of *S. epidermidis*, is primarily involved in cell wall turnover and cell division and lysis [45]. However, it was shown that AtlE-mediated cell lysis resulted in DNA release (extracellular DNA, eDNA), which, in turn, promoted the adhesion of the surrounding cells to the polystyrene surface [46]. Thus, AltE seems to mediate adhesion through the release of DNA rather than acting itself as an adhesin. Another mechanism for the generation of biofilm eDNA in *S. lugdunensis* involves the competence protein ComEB, presumably via active DNA secretion [47]. Homologous autolysins were reported in other CoNS species, such as *S. caprae* (AtlC) [48], *S. warneri* (Atl [49]), *S. saprophyticus* (Aas) [50], and in *S. lugdunensis* (AtlL) [51]. Aae, another autolysin/adhesin found in *S. epidermidis*, is as well implicated in the initial adhesion to abiotic surfaces [42,52]. The protein Aap also participates in the initial adhesion to abiotic surfaces [53,54]. This protein consists of an N-terminal Domain-A and a C-terminal Domain B, the Domain-A being the one involved in the adhesion to abiotic surfaces [53]. Other studies have suggested the involvement of ClpP [55], SdrF [56], and Bhp [57] in this process.

In respect to non-proteinaceous molecules, TAs play an important role in initial adhesion. TAs are anionic glycopolymers highly abundant in the cell wall that are involved in several essential cell functions (reviewed in [58,59]). TAs are divided into wall teichoic acids, which are covalently attached to the peptidoglycan layer, and lipoteichoic acids that are anchored to the plasma membrane. Both molecules are tailored with D-alanine esters by a process called D-alanylation [60]. This process balances the charge of both molecules. It was shown, in *S. aureus*, that mutants lacking the genes encoding the enzymes necessary to incorporate D-alanine into TAs resulted in a stronger negative net charge on the bacterial cell surface [61], thereby attenuating initial attachment to plastic surfaces [62,63]. Although TAs are less studied in CoNS, in *S. epidermidis*, the lack of wall teichoic acids resulted in impaired initial adhesion to polystyrene surfaces [64].

#### 2.1.2. Initial Adhesion to Abiotic Surfaces

When it comes to biotic surfaces such as living tissues or medical devices that are readily coated by host proteins after implantation, bacterial adhesion is facilitated by a different set of interactions, mostly by ligand–receptor specific interactions between host cells or extracellular matrix proteins and bacterial surface-associated adhesins. *S. epidermidis* and *S. aureus* express dozens of MSCRAMMs that bind to human matrix proteins such as fibrinogen, fibronectin, vitronectin and collagen, and often combine a binding capacity for several different matrix proteins [65,66]. As such, MSCRAMMs present a key function in initial adhesion to biotic surfaces, the role of SdrG being the best well-known. SdrG, also named Fbe, binds to its ligand, fibrinogen, by the “dock, lock, latch” mechanism. Studies performed to evaluate *S. epidermidis* SdrG function in the context of bacterial adhesion showed that this adhesin is important in vitro, not only to mediate bacterial binding to fibrinogen coated-surfaces [40] but for platelet adhesion and aggregation [67]. More recently, it has been suggested that SdrG can bind to host cells, such as osteoblasts [68]. SdrG has also shown to be important in vivo for the colonization of implanted material [69]. A homolog to SdrG, the fibrinogen binding protein (Fbl), was found in *S. lugdunensis* [70] and is likely to be involved in this bacterium initial adhesion to biotic surfaces.

Also involved in the adhesion of *S. epidermidis* to biotic surfaces are the proteins SdrF [71,72], SesC [73] and Embp [74], due to their affinity to, respectively, collagen and keratin, fibrinogen and fibronectin. In other CoNS additional adhesins with specificity to bind collagen were found, such as the SrdX in *S. capitis* [75], and the protein SrdI in *S. saprophyticus* that binds to both collagen [76] and fibronectin [77].

Besides the aforementioned role of the autolysins and TAs in the initial adhesion to abiotic surfaces, these molecules also present an important role in bacterial cells adhesion to biotic surfaces. The bifunctional autolysins AtlE and Aae, due to their affinity to vitronectin (AtlE and Aae), fibrinogen (Aae) and fibronectin (Aae) [46,52] and TAs because of their capacity to bind fibronectin [63,64] and adhere to epithelial and endothelial cells [78,79].

### 2.2. Maturation

After adhering to the surface, bacterial cells start dividing, forming aggregates and shaping its distinctive 3D appearance. To maintain a robust structure, cells need not only to be attached to a surface but also to stick to each other. As such, biofilm cells are embedded in a matrix composed of self-produced polysaccharides, proteins, lipids, eDNA and RNA, and TAs [80,81], but can also include molecules of the surrounding environment [82,83]. This extracellular matrix is fundamental for structural and functional roles as it provides stability against mechanical forces and creates a unique environment that is essential for the biofilm lifestyle [82,84]. Importantly, the matrix also plays a part in protection against disinfectants, antibiotics, immune cells activity, and bacteriophage (phage) predation [36,85,86]. Nevertheless, to ensure a functional organization, where nutrients are distributed into the deeper layers of the biofilm, channels need to be molded. To do so, disruptive forces need to be applied. Thus, during the biofilm maturation process, there is a thin balance between adhesive and disruptive forces [34].

#### 2.2.1. Intercellular Aggregation Accomplished by Adhesive Forces

In *S. epidermidis*, the most predominant adhesive molecule is the PIA, also named poly-N-acetyl glucosamine (PNAG) due to its chemical composition [87]. PNAG is synthesized by the enzymes codified by the intercellular adhesion (*ica*) locus, which is composed of the genes *icaA*, *icaD*, *icaB*, *icaC* [88], and the regulatory gene *icaR*, which is located upstream of the *icaADBC* and, thus, divergently transcribed [89]. In *S. lugdunensis*, even though *icaADBC* homologs were identified, the locus organization differs substantially from that of other staphylococci [90]. In addition, the *icaR* gene is absent even though another ORF was found in this position [90]. These differences may suggest an evolutionary adaptation that is likely to confer an advantage to this species [90,91].

Due to its proven fundamental role for biofilm structure, PNAG was for many years thought to be a requisite for biofilm formation. However, strains that did not harbor the *ica* genes were still able to form a biofilm, although less robust [92,93]. Thus, it was hypothesized that molecules other than PIA were implicated in biofilm maturation. We thus learned that biofilm formation can be supported or completely mediated by proteins [34,35,36]. In fact, *S. lugdunensis* biofilms are mostly composed of proteins rather than PNAG [90,93]. It was found that IsdC, an iron-binding protein, has a pivotal role in *S. lugdunensis* biofilm accumulation by promoting cells aggregation through homophilic interactions between IsdC molecules on neighboring cells [94].

Regarding proteins involvement in biofilm maturation, Aap is one of the best well-studied proteins in *S. epidermidis*. Biofilm accumulation by Aap is determined by Domain-B that becomes active only upon cleavage of the native protein [95,96]. Accordingly, the matrix of *S. epidermidis* biofilms is composed of a mixture of fully and partially cleaved proteins [35]. Recently, it was shown that the bacterial metalloprotease SepA is able to cleave the Domain-A of Aap resulting in enhanced biofilm accumulation in *S. epidermidis* [97]. Nevertheless, other unknown proteases, from either bacteria or the host, can cleave Aap, thereby contributing to biofilm accumulation. Aap promotes cell–cell adhesion by forming twisted rope-like structures through a Zn^2+^ dependent mechanism [98,99]. In addition, Aap is known to interact with N-acetyl glucosamine moieties potentially binding to PNAG, forming a protein-polysaccharide biofilm network [100]. Similarly, Embp seems to interact with PNAG contributing, this way, to the biofilm maturation, as, alone, it seems to be insufficient to create biofilm aggregation [74].

More recently, Sbp was also found to play an important role in *S. epidermidis* biofilm accumulation, having particular importance in the development of the biofilm architecture [101]. Sbp forms amyloid-like fibrils that function as a biofilm scaffold instead of directly inducing cell aggregation [102]. In addition, it was reported that Sbp interacts, through the fibrils formed, with the Domain-B of Aap also contributing to biofilm accumulation [102].

Other proteins such as SesC [73,103,104], SesJ [105], and SesI [106] were suggested to be involved in biofilm maturation. However, more studies are needed to undercover their relevance and mechanisms of action. Still, within the proteins domain, it is important to note that MSCRAMMs can also promote biofilm accumulation through homophilic interactions between MSCRAMMs in neighboring cells [107].

Lastly, as a result of their anionic character, both TAs [62,108] and eDNA originated from AtlE-mediated autolysis [109,110,111], can have accessory functions in aggregation by interacting with other surface polymers, via electrostatic interactions, thereby acting as a “glue”.

#### 2.2.2. Biofilm Structuring Accomplished by Disruptive Forces

As aforementioned, the disruption of the intercellular interactions is necessary for the formation of channels that ensure the passage of nutrients and waste in and out of the biofilm. In staphylococci, proteases [112], nucleases [113], and phenol-soluble modulins (PSMs) [114,115] have been implicated in this role. However, only PSMs have been consistently demonstrated to assist in biofilm structuring, both in vitro and in vivo (reviewed in [116]).

PSMs are amphipathic α-helical molecules with strong surfactant-like properties. As such, it is thought that PSMs contribute to biofilm structuring by disrupting non-covalent interactions that occur between biofilm matrix molecules [42]. *S. epidermidis* produces six PSM peptides: PSMα, PSMβ1, PSMβ2, PSMδ, PSMε, and PSMγ (δ-toxin) [117], which are encoded in the chromosome, and the PSM-mec that is encoded in the mobile genetic element SCC*mec* [118]. PSMβ peptides have been shown to be a key effector in biofilm structuring and dispersion both in vitro and in vivo [114]. A deletion mutant of the β-type PSMs developed a more compact and extended biofilm than the parental strain [114]. As could be expected, PSMβ also has a role in the biofilm dispersion phase, the last step of the biofilm lifecycle. It was shown that, depending on the level of production, PSMβ can lead to either biofilm structuring (medium concentrations) or biofilm dispersion (higher concentrations) [114]. The production of PSMs is strictly regulated by the accessory gene regulator (*agr*) quorum-sensing (QS) system, which will be discussed further in Section 2.4

### 2.3. Dispersion

As the biofilm grows older, cell clusters may leave the biofilm [119]. This is an important phase as it contributes to biofilm expansion, bacteria survival, and disease transmission [119]. While not as explored as the initial adhesion or biofilm maturation, the dispersion step is a complex process having drawn some attention in past years, in particular, in oral bacteria and *Pseudomonas aeruginosa*, with only a few studies performed in staphylococcal species.

Currently, the dispersion phase is divided into two mechanisms, which are defined based on the initial trigger: (i) passive dispersion, also called detachment, which includes processes mediated by external factors, and (ii) active dispersion, which integrates processes actively employed by bacteria in response to external signals [120]. Passive dispersion can occur by several different mechanisms such as abrasion (removal of cells due to collision with particles), grazing (due to the activity of eukaryotic predators), erosion, and sloughing (removal of cells or larger pieces of the biofilm by fluid shear) (reviewed in [119,120,121]). Also in this category are the techniques developed to induce detachment such as enzymes with the capacity to degrade biofilm matrix macromolecules (mainly polysaccharides and proteins) and physical biofilm disruption [119]. One of the best well-known enzymes with the capacity to disperse *S. epidermidis* biofilms is Dispersin B [122], a PNAG-degrading enzyme produced by *Actinobacillus actinomycetemcomitans* [123]. To what concerns the active mechanisms of dispersion and the effector molecules associated, as detailed in Section 2.2.2, PSMs, in particular, β-type PSMs, are the major players.

Even though a lot of research has been focused on the characterization of biofilm cells phenotype, very little is known about the cells released from the biofilm. Initially, it was hypothesized that, after leaving the biofilm, cells would immediately revert to their planktonic phenotype [112]. However, later on, other studies have demonstrated that cells released from biofilms present a particular phenotype, although transient, that is different from both planktonic and biofilm cells [124,125]. In *S. epidermidis*, the cells released from biofilms present a higher tolerance than biofilm or planktonic cells to some antibiotics [126] and elicit a more pro-inflammatory response in a murine model of hematogenous disseminated infection [127]. Nevertheless, more studies are necessary to further understand the mechanisms behind biofilm dispersion and the role of the cells released in the virulence of CoNS.

### 2.4. Regulation of Biofilm Formation

To form the complex structure displayed in biofilms, bacteria have to tightly coordinate every single step of the process. As such, there are several regulatory systems involved in biofilm formation by staphylococcal species, the *agr* QS system being one of the best characterized (reviewed in [37,128,129]).

Shortly, the *agr* system is a classical two-component signaling system that is activated by an autoinducing peptide (AIP) when this reaches a critical concentration, i.e., “quorum” cells in the population. This signal is sensed by bacteria that synchronize their response. The *agr* locus codifies the RNAII and RNAIII transcriptional units that are regulated by two different promoters, respectively, P2 and P3. The RNAII transcript encodes the genes *agrBDCA* and the RNAIII the *hld* gene that is responsible for the production of the PSMγ (δ-toxin) [130]. Interestingly, although *S. lugdunensis* holds an *agr*-like system, the *hld* gene is encoded elsewhere [131,132]. Mechanistically, the signaling cascade starts with *agrD*, which is post-translationally modified and exported by AgrB. The extracellular accumulation of the AIP is detected by the histidine kinase AgrC that, in turn, activates the DNA-binding regulator AgrA. This activates P2 and P3 promoters [130,133], as well as the ones controlling the expression of α- and β-type PSMs transcripts [134]. Lastly, RNAIII, the effector of the *agr* system, directly controls the upregulation of genes encoding enzymes, toxins, and PSMs, and it downregulates several genes encoding surface-associated adhesins [135]. This regulation occurs either by modulating transcription initiation or at the post-translational level by interacting with the target gene transcript [133].

Probably related to *agr* system downregulation of adhesins and upregulation of PSM and other proteases, the dysfunctionality of the *agr* system in *S. epidermidis* results in thicker biofilms with defects in dispersion capacity [32,136]. Although one may think that the *agr* negative phenotype is not advantageous as it impairs the bacterium capacity to disseminate, this phenotype is frequently seen in bacteria isolated from catheter-related infections. This suggests that mutations in the *agr* system have an adaptive advantage to cause IMD-associated infections [32], possibly because a thicker biofilm is likely to confer fitness advantage in chronic infections [32,137,138]. It was proposed that the naturally occurring mutations in the *agr* system are likely to be related to the high metabolic burden that the maintenance of the *agr* system poses to the cell [129].

A second QS system molecule influencing biofilm formation in CoNS is the autoinducer-2 (AI-2) that belongs to the LuxS/*AI-2* QS system. Due to its wide distribution in many bacterial species, this seems to be an interspecies communication system [129]. AI-2 controls biofilm formation by positively regulating the *ica* operon repressor *icaR*. In *S. epidermidis*, the absence of AI-2 resulted in higher expression of PNAG and, consequently, increased biofilm formation [139]. In addition, the absence of AI-2 led to increased virulence in central venous catheter-associated infection model [139]. It is important to mention that studies performed with other *S. epidermidis* strains reported a contradictory effect, where AI-2 leads to icaR negative regulation [140].

Biofilm formation can also be regulated by different factors such as Sigma B (SigB) and the staphylococcal accessory regulator A (SarA). Sig B is an alternative sigma factor of RNA polymerase, which leads to global changes in gene expression when activated by stressful situations. The lack of SigB in *S. epidermidis* resulted in increased expression of *icaR*, which repressed the production of PNAG and, consequently, impaired biofilm formation [141,142]. In addition, the disruption of SigB production led, in *S. epidermidis*, to impaired colonization in a catheter-associated infection model [143]. Lastly, SarA is a general transcription factor that binds to AT-rich sequences, activating or repressing the expression of the target genes [129]. Nevertheless, the effect of SarA in *S. epidermidis* biofilm formation is highly strain-dependent. While in some strains SarA mutation led to a biofilm-negative phenotype through the downregulation of *ica* operon expression by an IcaR-independent pathway [144], in *aap*- and *ica*-negative strains resulted in higher biofilm formation capacity through the overexpression of the protein Empb and release of eDNA by a SepA and AtlE-mediated process [145].

Additionally, although not a true regulator, the insertion of the insertion sequence (IS)256 in *ica* genes abolishes PNAG production [146].

## 3. Persistence as a Tolerance Mechanism

Bacteria can quickly respond to unfavorable environmental or stressful conditions by lowering their metabolic activity, altering their gene expression, or by inducing genetic changes, entering a state of dormancy [147]. Biofilms per se are an example of a bacterial stress condition, namely due to nutrients and oxygen deprivation [148]. Biofilm-embedded communities are characterized by the presence of heterogeneous cells, with distinct physiological states, whose emergence depends on the micro-environmental conditions in its surroundings [149,150]. Therefore, since the access to nutrients and oxygen at the deeper biofilm layers is more unfavorable than in the upper layers, variant subpopulations of cells can emerge [151]. Importantly, CoNS can switch to a different mode of growth and adjust their gene expression patterns, metabolic activity, and phenotype, to promote their survival in stressed or limited environmental conditions [152,153,154]. Recently, two CoNS species, namely *S. epidermidis* and *S. haemolyticus*, exhibited different strategies to overcome the impact of nutrient depletion [155]. While *S. epidermidis* managed to survive through the accumulation of cardiolipin and/or lyso-cardiolipin, *S. haemolyticus* employed a completely different strategy, surviving the nutrient depletion created by building an extremely simple lipidome, made of only diglucosyl-diacylglycerol and phosphatidylglycerol. Additionally, the authors claimed that bacteria at the stationary phase seemed to have similar behavior as when exposed to starvation [155]. Considering the entrance of bacteria into dormancy upon stressful conditions (e.g., starvation), the analysis of bacteria in the stationary phase may highlight some potential strategies used to survive those environments. Some dormancy phenotypes have been already found and will be detailed below.

### Bacterial Cells Dormant Phenotypes: A Tolerance Mechanism

Several authors have been debating the possible different phenotypic states that bacteria can undertake, which enable a diminished inflammatory response and higher tolerance to the antimicrobial therapies applied [156,157,158]. Persisters, VBNC, and SCVs are the physiological states currently under debate.

In the 1940s, persister cells were described and defined as a group of cells that exhibit a drug-tolerant phenotype [159,160]. This small subpopulation of cells, when exposed to antibiotic pressure, becomes slow-growing by reducing their metabolism, rather than promoting an active response. Once the stress is removed, persister cells can resume growth, contributing to the antibiotic tolerance observed among biofilms cells [161,162]. The formation of persister cells in culture can be reached through two distinct ways: triggered or spontaneously. Triggered persisters, also defined as type I persisters, emerge when cells encounter some stress, such as starvation, and the persistence level may depend on the type and the intensity of the trigger [161]. Spontaneous persisters (type II persisters) occur during the stationary phase culture and persist as long as the steady-state growth is maintained [161,163,164]. The presence of persisters can be found on both planktonic and biofilm populations, as observed in *S. epidermidis* cells when exposed to levofloxacin and vancomycin [165]. Goneau et al. were able to induce the formation of persister cells in *S. saprophyticus* using antibiotics from different classes (ciprofloxacin, ampicillin, and gentamicin), exhibiting a greater antibiotic tolerance during the stationary phase than in the exponential phase [166]. A recent study on CoNS demonstrated the formation of persister cells after exposure to various biocides (polymyxin, sodium sulfacetamide, lysing solution). Independently of the biocide tested, *S. epidermidis* and *S. capitis* strains were able to form persister cells [167].

Later in 1982, the existence of another phenotype, viable but non-culturable cells, was proposed. These cells were identified by Xu et al., which showed that these bacterial cells could not grow on routine or selective media [168]. Moreover, VBNC cells were discriminated from dead cells, since they were similar to live cells, containing an intact membrane, an active mRNA transcription, metabolic activity, and respiration [169,170]. At least eighty-five bacterial species have been shown to enter a VBNC state, including foodborne and clinical pathogens [171]. To date, *S. epidermidis* is the only known CoNS reported to adopt this survival strategy [172]. Cerca and co-workers have developed a model where the proportions of VBNC cells in *S. epidermidis* biofilms can be modulated. Briefly, the authors demonstrated that the induction of VBNC cells could be achieved by increasing the glucose concentration in the growth medium and that this induction could be somehow prevented by the supplementation of the medium with Ca^2+^ and Mg^2+^ [172]. Several methods have been suggested to uncover the existence of VBNC cells. Assessing viability and culturability is the key to provide an estimation of the number of these cells [173]. Therefore, numerous approaches have been combined to evaluate cell viability and culturability, such as the usage of fluorescent microscopy or flow cytometry and colony forming units (culture methods), respectively [174,175]. Additionally, a study demonstrated that the combination of LIVE/DEAD staining with quantitative PCR can also reveal the presence of VBNC cells in CoNS biofilms [175]. Over the years, the similarities and/or differences between persisters and VBNC cells phenotypes have been a motive for intense debate since both are employed by bacteria under the same stress conditions. Some authors suggest that persister cells are indeed VBNC cells [176,177], while others oppose this interpretation as the similarities hypothesized for both phenotypes were described in different species [178]. Moreover, it has been suggested that persister cells are more associated with antibiotic stress and can easily regain growth after antibiotic removal, whereas the VBNC state seems to be linked to different environmental conditions and, in some species, the removal of the stress factor is not enough to revert the phenotype, requiring a more specific condition to revive the cells [178].

SCVs were first described more than 100 years ago [179]. These cells are known as natural occurring bacterial subpopulations, demonstrating a similar slow growth rate as the previously described dormant phenotypes and, as the name implies, exhibit a smaller size than their parental wild-type bacteria, setting a challenge in their identification [180]. Since then, SCVs were found in a wide range of bacterial species such as *S. aureus* [181] and CoNS species and are generally correlated to biomaterial-associated infections [180,182]. Several aspects of the pathogenic potential of SCVs have been described, mainly their enhanced biofilm-forming ability [183], evasion from the immune system response [184], and their resistance against antimicrobial agents [185,186]. Interestingly, Onyango et al. revealed that *S. epidermidis* and *S. lugdunensis* were capable of developing SCVs phenotypes following the exposure to a wide range of environmental stress conditions, such as pH alterations (pH5), osmotic stress (0–20% NaCl), low temperature (4 °C), and to the presence of antimicrobial agents (vancomycin and penicillin G) [187]. Additionally, the authors found a thicker extracellular matrix in all SCVs populations in comparison to their corresponding control cells [187]. Therefore, this feature may represent an adaptation in biofilm formation to provide a stronger defense against antimicrobial agents, as suggested by other authors [188].

However, the genetic bases underlying these dormant phenotypes are still not well characterized. Therefore, it is important to recognize the impact of these dormant phenotypes, as a tolerance mechanism adopted by CoNS, in nowadays clinical infections and food safety.

## 4. Antibiotic Resistance

The significance of CoNS species has increased over the years, mainly due to their multidrug resistance profile [24,189,190,191,192,193] and their ability to grow as biofilms, which are even more refractory to antibiotics as reported worldwide [31,191,194,195,196,197]. Several mechanisms have been discussed concerning the increase of antimicrobial resistance, from which (i) the barrier formed by the matrix surrounding the cells within biofilms, whose thickness and composition can hinder the penetration and/or diffusion of antibiotics [198], and (ii) the fact that staphylococci biofilms are very prone to mutations that may increase their resistance towards antibiotics [199,200] stand out. Moreover, the presence of cells with distinct physiologies, such as persister, SCVs and VBNC cells, also increases tolerance to antibiotics [201,202] (reviewed in [203,204,205]). Another possible explanation for the high rate of antimicrobial resistance is that CoNS share the same niches of colonization with *S. aureus*, allowing horizontal gene transfer (HGT) of several genes and mobile elements encoding for antibiotic resistance [206]. In fact, HGT among staphylococcal species has already been proven with the detection of many resistant phenotypes related to multiresistant genes located on mobile genetic elements [25,207,208]. The importance of mobile genetic elements as virulence factors in CoNS will be further explored in more detail in Section 5.

Fighting these threats has hence become of ultimate importance, with the main strategy being the application of a cocktail of several antibiotics for a prolonged period of time [24,209,210,211,212,213]. However, the high tolerance of CoNS biofilm cells commonly causes the failure of antibiotics, even when the most severe therapies are used [214] and, in the cases associated with the use of IMD, may implicate the removal of the infected device, resulting in prolonged hospital stays and increased morbidity and mortality rates [42,203,215]. Interestingly, the problematic of IMD-associated infections was emphasized by a recent study that assessed *S. epidermidis* in-host evolution in a case of pacemaker-associated endocarditis, which has shown that increased tolerance to antibiotics and capacity to form biofilms occurred during the course of infection [216]. This helps to explain the often inefficacy of antibiotics to treat *S. epidermidis* infections. Besides the impact on human health, CoNS infections and contamination are also alarming from the veterinary and food production standpoint, where antimicrobial resistance has correspondingly been reported (reviewed in [14,24,217,218]). Among CoNS strains, antimicrobial resistant rates have been increasing over the years, resulting from (i) the incorrect and/or widespread use of antibiotics, (ii) the use of antibiotics in domestic and farm animals, (iii) the low discovery rate of newer antibiotics, and (iv) due to the intrinsic environmental conditions contributing to the adaptation of bacteria to antimicrobial compounds [219,220,221,222,223,224,225,226].

### 4.1. Resistance to β-lactams

Some of the more representative species of CoNS are known to present a high resistance rate to methicillin [25,227], as, for instance, *S. epidermidis* [228], *S. haemolyticus* [229], and *S. sciuri* [230]. This phenotype is not region-specific as studies from Europe to North America have shown that 60 to 80% of the CoNS species retrieved from bloodstream infections were resistant to methicillin (MR-CoNS) [231,232,233,234]. Not surprisingly, such isolates often present increased tolerance to most β-lactam antibiotics, whose structure and mechanism of action are similar to methicillin [235,236]. The resistance to the action of β-lactamase was first described as the result of the hydrolysis of the β-lactam ring of such antibiotics, by penicillinases [237], as determined by the plasmid-mediated staphylococcal β-lactamase *bla-Z* [238]. Now, it is known that staphylococcal species can produce a specific penicillin-binding protein (PBP2a), which is responsible to completely inactivate the activity of most β-lactams, and that this resistant phenotype is complex and related to the existence of SCC*mec*, a staphylococcal cassette chromosome containing the *mecA* gene, which encodes the PBP2a protein [239,240,241]. Importantly, already back in the 1980s, it was found that only about 10–20% of CoNS isolated from nosocomial infections were penicillin-susceptible, contrary to the 80% of commensal isolates being susceptible to methicillin [242,243]. Thisseems to remain true in the present days, where more than 90% of CoNS isolated in the hospital settings present increased resistance to penicillin-derived antibiotics [222,229,244].

### 4.2. Resistance to Other Antibiotics

Over the years, there has been an increase in the number of CoNS strains resistant to glycopeptides, which are the antibiotics often used to treat MR-CoNS infections, as well as the emergence of resistance to newer antibiotics, hindering the current treatment options. For instance, *S. epidermidis* was shown to be resistant to up to eight distinct antibiotics with different mechanisms of action and it is estimated that, among nosocomial isolated strains, 80% of the isolates present resistance to antibiotics beyond methicillin [232,245]. Contrasting with previous studies with a broad range of CoNS, where few isolates and species presented increased tolerance to antibiotics like vancomycin and teicoplanin [246,247,248], the emergence of isolates with reduced susceptibility to glycopeptides has been reported in several species (Table 1). Surprisingly, resistant isolates of *S. epidermidis* [249,250] and *S. haemolyticus* [251,252] were detected already three decades ago. *S. warneri* [253,254,255] and *S. capitis* [256,257] have also joined the list with several isolates resistant to vancomycin, generating outbreaks especially in neonatal units, where *S. epidermidis* resistant isolates are also frequently found [258]. Fortunately, vancomycin remains an effective antibiotic against most of the CoNS isolates [189,222,245,259], being on the top list of antibiotics used to fight these infections, either alone or in combination with other antibiotics as cefazolin [209], rifampicin [213,260], and fosfomycin [261], among others. Rifampicin is also frequently used to treat staphylococcal infections; however, this antibiotic is associated with the rapid development of resistance when used alone and, as such, it should be used as part of a combined therapy [262,263,264]. For instance, the use of vancomycin or levofloxacin with rifampicin has been proved to be a good combination to treat these infections [213]. Significant and concerning increases in the resistance to ciprofloxacin, clindamycin, erythromycin, gentamicin, and tetracycline have been found over the last few years [22,191,219,265,266,267,268,269,270]. Resistance to tetracycline is commonly based on the acquisition of mobile resistance genes that lead to the dissociation of tetracyclines from their ribosomal binding sites and transportation of the antimicrobial agents out of the cell through drug efflux pumps [271,272]. Linezolid belongs to a newer class of antibiotics (oxazolidinones) and appeared as a promising alternative to fight staphylococcal infections with multi-drug resistance to common antibiotics [273,274], including glycopeptides, to which bacteria have already developed resistance mechanisms. Nevertheless, resistance to linezolid has already been reported in staphylococci including CoNS [275,276]. Another antimicrobial belonging to the next-generation antibiotics is daptomycin, which has proven to be more effective than vancomycin against MR-CoNS [277]. Despite being a new antibiotic, there are already reports of isolates resistant to daptomycin [278], hence, the combination therapy with other antibiotics as rifampicin [279] may be suggested.

### 4.3. Antimicrobial Resistance in the Community

Despite most of the studies being focused on clinical strains, it is known that staphylococci isolated from healthy individuals may also present increased antimicrobial tolerance. In fact, several studies report the carriage of distinct CoNS antibiotic-resistant commensal strains by the community [195,341,342], even in remote populations [227]. Although community strains may present lower resistance rates, as, for instance, only up to 20% of *S. epidermidis* commensal strains were found to be resistant to methicillin [343,344], contrasting with approximately 80% of resistance found among clinical isolates [245,345,346], the main existence of commensal strains with antimicrobial resistance colonizing humans and other mammals is alarming [347]. This is especially concerning in immunocompromised individuals, as CoNS are often considered opportunistic pathogens that may cause severe infections, whose treatment would be hindered by the existence of isolates with antimicrobial resistance, as reviewed by Heilmann et al. [8]. The presence of CoNS with increased tolerance or resistance to antibiotics in animals and food is also worrying. Several studies report the isolation of CoNS with multidrug resistance recovered from bovine mastitis [14,348], retailing chicken meat [349], livestock, bulk tank milk, and minced meat [25], as well as from ready-to-eat foods [23]. Undoubtedly, the presence of antimicrobial-resistant strains in animals represents a challenge to the animal hosts, as infections become harder to treat, but may also be problematic to human hosts upon transmission of resistant strains resulting from close contacts between people and companion or farm animals [208,220,221].

## 5. Mobile Genetic Elements

As mentioned before, CoNS infections are associated with the establishment of biofilms. It is actually in these complex structures that HGT phenomena are favored due to high cell density, high genetic competence, and availability of mobile genetic elements [350]. HGT is a highly important force driving bacterial evolution. Bacterial adaptation to new niches and environments frequently occurs through the acquisition of new genes by HGT processes. There are three different mechanisms to which HGT can occur: (i) transformation, (ii) transduction, and (iii) conjugation. Transformation occurs when a DNA fragment from a dead or compromised cell enters a competent bacterial cell. Transduction consists of the transfer of DNA between bacterial cells through a phage. Although some lytic phages can transduce, generally temperate phages are more frequently associated with this HGT mechanism. Under the lysogenic cycle, the viral genome is integrated into the bacterial chromosome establishing a prophage. Under certain stimuli, phage genomes are excised from the bacterial genome, and, occasionally, they exchange a small piece of bacterial DNA for a piece of the phage genome. The newly formed phages are then composed of these DNA regions that can be further inserted in new bacterial cells upon a new cycle of infection. Conjugation is the transfer of DNA directly from one cell to another through cell–cell contact. This process usually involves the transfer of plasmids. High genetic relatedness has shown to be a key factor influencing HGT because, as phylogenetic distance increases, HGT phenomena diminish [351]. As phage propagation depends on host genetic similarity, transduction usually just occurs throughout the same species or genus [352]. In opposition, plasmids and integrative and conjugative elements can cross the interspecies barrier [353].

The HGT mechanism also influences the size of the nucleotide sequence that is transferred. While on phage-mediated transduction up to 45 kb chromosomal DNA or plasmids (small- or middle-sized) are transferred, larger plasmids are transferred through conjugation [354,355]. Moreover, in 2016, Haaber et al. discovered a mechanism named autotransduction in which phages are spontaneously released from the bacterial chromosome, and, after infecting a susceptible cell, they transfer DNA from this cell to the lysogenic population [356].

In the last decade, it was evidenced that CoNS might act as reservoirs of genes that can be transferred between different staphylococci, having the potential to increase the virulence of several species, namely *S. aureus* [357,358]. Indeed, genes conferring resistance to all classes of antibiotics observed in CoNS are usually located on mobile genetic elements [359]. Staphylococcal plasmids have been shown to confer resistance to numerous antibiotics, namely tetracyclines, macrolides, amphenicols, and aminoglycosides [360]. Different strains containing these plasmids have been isolated from different environments, namely hospitals, veterinary, and effluents [361,362,363]. Despite being described as highly variable, the same staphylococcal plasmids have been shown to be widely geographically distributed [354]. This high similarity of plasmids, structure, and gene content suggests that they are transferred horizontally between strains in different environments [359].

Other key conjugative elements in staphylococci are the integrative conjugative elements (ICEs), from which two main families have been recognized both in CoPS and CoNS [22,355]. The transposon Tn916 and the integrative conjugative element ICE6013 [364]. Tn916 includes the well-studied Tn5801 subfamily that encodes tetracycline resistance and a protein that can inhibit restriction barriers of incoming DNA (when heterologously expressed in *Escherichia coli*) [365,366]. ICE6013 was first discovered in *S. aureus* and described to have 15 ORFs, the shortest being known as the ICE [355]. So far, seven subfamilies of ICE6013 were identified with an Average Nucleotide Identity (ANI) of 68–79% between them [364]. This element uses, as recombinase, an IS30-like transposase that offers versatility on the integration sites.

Usually, in bacterial genomes, there are specific regions that are flanked by direct repeats, named genomic islands (GI), that are usually acquired through HGT events. On a sensu lato, the GI concept encompasses all elements with mobility functions, namely ICEs, integrative mobilizable elements (require helper functions to conjugate), and transducible elements (SCC elements and pathogenicity islands) [367]. These GI can vary in size and are classified based on the products their genes encode. An important example is the SCC*mec*. This element carries the *mecA* gene, which is responsible for the resistance to methicillin and other functional genes, namely the cassette chromosome recombinase (*ccr*) genes that encode recombinases responsible for mediating the integration and excision of this element into and from the bacterial chromosome. Furthermore, SCC*mec* comprises transposons, insertion sequences, and plasmids [368]. The presence of this element is of great importance as it provides resistance to all penicillin-like antibiotics. Currently, in staphylococci, 11 different SCC*mec* types were described (*I*–*XI*). It is important to highlight that, while some SCC*mec* types only encode resistance to β-lactam antibiotics, others can confer resistance to several antibiotics, as they contain transposons or integrated plasmids [369]. There is clear evidence that *S. epidermidis* can act as a reservoir of SCC*mec*, suggesting HGT events between CoNS and *S. aureus* [370].

Phage-mediated transduction events also occur on the pathogenicity islands dissemination. In *S. aureus*, they are known as SaPIs—*Staphylococcus aureus* Pathogenicity Islands. The movement of pathogenicity islands occurs through the use of helper phage capsids. Usually, SaPIs encode phage-like proteins that facilitate the transfer process, such as a repressor (Stl) that controls SaPI excision and interaction with the helper phage. It is important to highlight that these interactions depend on the phage and the SaPI [371]. Although SaPIs typically encode one or more virulence determinants, they are rarely composed of antibiotic resistance genes. Nevertheless, SaPIs are composed of large variable regions that can be acquired, modified, or even removed. Despite these regions being widely studied in *S. aureus*, some orthologous regions were already identified in CoNS. Pathogenicity islands were already found on the *S. epidermidis* FRI909 strain. Although initially this strain was referred to as an *S. aureus* strain, it was further reclassified as *S. epidermidis* [372]. This SePI (*Staphylococcus epidermidis* pathogenicity island) is composed of two regions separated by repeat motifs and encodes the staphylococcal enterotoxins *sec* and *sel* [373].

Recently, Banaszkiewicz et al. analyzed more than 1500 staphylococci genomes and found out that five *S. epidermidis* strains contained the same number of ORFs (*n* = 29) as *S. epidermidis* FRI909 [374]. Moreover, several other strains were only missing a small number of these ORFs. The authors concluded that these SePI-associated elements present in *S. epidermidis* can be related to the acquisition of virulence-associated genes, suggesting that gene exchange between *S. aureus* and CoNS can lead to the emergence of new highly pathogenic *S. epidermidis* strains [374].

As stated before, phages are involved in HGT by being responsible for transduction events. The majority of bacterial species contain prophages in their genomes, being responsible for an important genetic variability [375]. Throughout their genomes, prophages encode a set of genes that can contribute to bacterial virulence or fitness [376]. For the referred reasons, phages play an essential role in bacterial evolution and adaptation. Regarding staphylococci prophages, the vast majority of the studies are performed with *S. aureus* [377,378,379,380]. Several phage-encoded virulence factors have been described for this pathogen, for example, Panton–Valentine leukocidin, exfoliative toxin A, enterotoxin S, staphylokinase, and the staphylococcal complement inhibitor [379]. Moreover, it has also been described that, through negative lysogenic conversion, phage integration can disrupt the expression of host-encoded virulence genes [381]. Despite the number of prophages observed in CoNS genomes being lower than in *S. aureus*, it has been suggested that phages might also be involved in the pathogenesis and evolution of CoNS [382]. The majority of the staphylococcal prophages belong to the *Siphoviridae* family. Prophages were already described on several species, namely *S. epidermidis*, *S. carnosus*, *S. hominis*, *S. capitis*, and *S. haemolyticus* [383,384,385,386,387]. Regarding genomic structure, CoNS and *S. aureus* prophages are very similar. An important difference between them is that the majority of the virulence factors observed in *S. aureus* are absent in CoNS prophages. The close relationship observed between staphylococci prophages may increase the probability of prophage-mediated HGT between different staphylococcal species [384]. Generally, it has been speculated that mobile elements have transferred from CoNS to *S. aureus* [358]. Clustered Regularly Interspaced Short Palindromic Repeats (CRISPR) elements found in CoNS isolates determine incorporation of foreign DNA into the genome and may limit the acquisition of mobile genetic elements, including enterotoxin genes [34]. Consequently, we might expect the unidirectional acquisition of mobile elements from CoNS by *S. aureus*. This evolutionary scenario explains the acquisition of the SCC*mec* cassette, Arginine catabolic mobile element (ACME), and *sasX* genes from CoNS [388,389]. However, the distribution of CRISPR elements is much lower in *S. epidermidis* and other staphylococci than previously thought and there is recent evidence of frequent HGT and exchange of mobile genetic elements within and between staphylococcal species [390,391]. *S. aureus* may even act as a source of mobile elements for CoNS, including pathogenicity island exchange, as demonstrated by the transduction of *S. aureus* SaPI to *S. xylosus* and *S. epidermidis* [392,393].

## 6. Bacteriocins

Due to the highly competitive and polymicrobial environment that bacteria live in, they have developed several defense mechanisms for self-preservation. These mechanisms include, among others, the production of molecules with quorum quenching ability [394,395], exotoxins [396,397], antibiotics [398], and bacteriocins [399,400].

Bacteriocins, defined as ribosomally synthesized peptides with antibacterial properties [401], are one of the most widely distributed microbial defense mechanisms. Indeed, it is estimated that 99% of bacteria produce at least one bacteriocin [402]. These molecules allow the producer to outcompete the competitors in its surroundings, to invade new and established niches and, ultimately, can modulate the composition of the involving microbiota [403,404]. Both Gram-positive or -negative bacteria have the capacity to produce bacteriocins, but the vast majority reported so far are produced by the former [405,406]. Due to the great diversity of bacteriocins produced, their classification has been a motive of controversy, having classes/subclasses been proposed and withdrawn over the years. One of the most comprehensive and straightforward classification systems categorizes the bacteriocins produced by Gram-positive bacteria into four classes [407]. Class I bacteriocins comprise small peptides (<5 KDa) that go through extensive post-transcriptional modifications containing, thus, unusual amino acids in its composition. Class II also comprehends small peptides (<5–10 KDa) but without or with minor post-transcriptional modifications. Class III includes large proteins (>10 KDa) and class IV comprehends complex bacteriocins that are conjugated with lipids or carbohydrates moieties [407]. More detailed information about the structure, characteristics, properties, and modes of actions of each class of bacteriocins produced by Gram-positive bacteria, in particular produced by CoNS, are comprehensively and recently reviewed elsewhere [408,409]. Although initially thought to only target closely related bacteria, some bacteriocins have a broader spectrum of activity affecting bacteria across different genera [410] or even transphylum [407]. Besides presenting a variable spectrum of activity, their high diversity [411], high stability at elevated temperatures and wide range of pH [412,413], relatively low cytotoxicity [407,414], and amenability to bioengineering [410] render bacteriocins interesting for an array of applications in food, agriculture, veterinary, cosmetics, and pharmaceutical industries (reviewed in [415,416,417]).

Considering that the production of bacteriocins is triggered by the surrounding competitors, CoNS being found in a variety of environments and hosts [418] they produce bacteriocins with far-reaching activity targeting pathogens that affect foodstuff, plants, animals, and humans (reviewed in [409]). The majority of the bacteriocins produced by CoNS are lantibiotics [400], which are characterized by harboring the unusual and non-proteinogenic amino acids lanthionine and 3-methyllanthionine [419]. *S. epidermidis* is known to produce several lantibiotics such as Pep5 [420], epidermin [419], epicidin 280 [421], epilancin k7 [422], epilancin 15× [423], and nukacin IVK45 [424]. Likewise, other CoNS produce other important lantibiotics such as gallidermin (*S. gallinarium*) [425], hominicin [426] and nukacin KQU-131 [427] (*S. hominis*), nukacin ISK-1 [428] and SWLP1 [429] (*S. warneri*), nukacin 3299 [430] (*S. simulans*), and Nisin J [431] (*S. capitis*). However, CoNS also produce bacteriocins belonging to other classes such as the epidermicin NI01 (produced by *S. epidermidis*) [432] and capidermicin (*S. capitis*) [433], which belong to class II, and endopeptidade ALE-1 (*S. capitis*) and lysostaphin (*S. simulans*) [434] that belong to class III bacteriocins. Because (i) several staphylococcins target clinically important pathogens such as *S. aureus*, including methicillin-resistant and vancomycin-intermediate, (ii) the shortage in novel and efficient antibiotics, (iii) the increase in antibiotic resistance, and (iv) the lower toxic effect of bacteriocins when compared to antibiotics [406], the potential use of staphylococcins against both human and animal pathogens has been particularly explored (reviewed in [408,409]). Despite the promising results obtained in vitro, both in planktonic and biofilm modes of growth [426,431,433,434,435,436], only a few staphylococcins, namely lysostaphin [437,438,439] and epidermicin NI01 [440,441], were evaluated using in vivo models. These two bacteriocins constitute promising candidates as therapeutic antimicrobial agents, lysostaphin currently being in late clinical trials for topical application [409].

As a result of their natural origin and consumers demands for products with no chemical additives, the interest for bacteriocins in food preservation has increased. Several bacteriocins, mainly produced by lactic acid bacteria, have been used in food industries for many years already [442,443] being Nisin, planctaracin, sakin P, and pediocin the most commonly used and commercially available [442]. While not as explored, the application of the staphylococcins in food processing environment is encouraging since, as mentioned before, several bacteriocins produced by CoNS target *S. aureus* strains, which are one of the most important causative agents of food poisoning [444]. Indeed, recently, it was reported that pep5 and lysostaphin showed a remarkable capacity to reduce (between 95% and 99.99%) the load of enterotoxigenic *S. aureus* strains in cheese samples [445].

Despite its advantages and efficacy, substantial use of bacteriocins in the large scale industry was not yet conquered. This is mainly due to the difficulty to obtain practical quantities of its pure form and due to the high costs associated with its production and purification. While for food application partially purified and even crude preparations may be used, for clinical applications, pure bacteriocins are necessary [409]. As such, the biotechnological application of bacteriocins, as well as staphylococcins, has been delayed. However, since bacteriocins constitute excellent candidates to substitute antibiotics, the scientific community has not given up on bacteriocins just yet. As a result, to improve bacteriocins usage, research has shifted to a new paradigm, bacteriocins molecular engineering, to create variants of natural bacteriocins with improved solubility, stability, efficacy, pharmacokinetics, and to overcome the production and purification issues [403,446,447,448].

## 7. Impact on Health

The vast majority of infections caused by CoNS only rarely develop into life-threatening diseases. However, due to the variety of infections, their high frequency, and because they are extremely difficult to diagnose and treat, the infections caused by CoNS represent a serious burden for the public health system and, more importantly, have serious consequences on patients’ quality of life. The major risk factor for the development of infections with CoNS is the presence of IMD. These are essential for monitoring the patient’s vital functions, diagnosis, delivery of nutrients and/or drugs, and to support or replace failing organs [449]. As such, every year, millions of devices are used in industrialized countries [450,451,452]. However, while essential, IMD also provide a way into the human body and serve as a scaffold for biofilm formation by CoNS, these being capable of forming biofilms on a plethora of IMD (reviewed in [8,418,450,453] ). Furthermore, patients’ clinical conditions is another important risk factor, the most prone to develop infections caused by CoNS being the ones with immature or fragile immune systems such as preterm new-borns, elderly patients, patients with leukopenia, neutropenia, going through immunosuppression treatments, transplantation, chemotherapy, and care in intensive and burn care units [8,450]. With an increasing number of vulnerable patients, it is predicted that the number of people who can benefit from implantable devices will continue to rise. As a result, CoNS infections associated with the use of IMD will tend to rise, putting millions of patients at risk and an enormous economic pressure on healthcare systems.

### 7.1. Infections Caused by CoNS

CoNS are a very heterogeneous group having only a few species been regularly implicated in human infections (reviewed in [8,418]). In addition, there are differences regarding CoNS pathogenicity, having species that are considered completely innocuous, such as *S. carnosus*, other that display a medium-pathogenic profile as *S. epidermidis* and *S. haemolyticus* and, finally, others that are considered more virulent such as *S. lugdunensis* [27].

With regard to IMD-related infections, *S. epidermidis* is by far the most representative species of the group, followed by *S. haemolyticus*, *S. hominis*, and *S. saprophyticus* [8]. Bloodstream infections (BSI) are the most common outcome of CoNS colonization of medical devices and are especially associated with the use of intravascular catheters or implant ports [454,455]. Moreover, BSI can also arise from the colonization of other types of devices, for example, prosthetic heart valves [456], cardiac assist devices [457,458], and coronary stents [459]. Of note, BSI caused by CoNS independently of the use of medical devices can also occur, mainly affecting preterm newborns [460] and neutropenic patients [461]. BSI symptoms can be subtle and nonspecific at the beginning but may lead to severe complications and a fatal outcome being, thus, a major concern within the infections caused by CoNS infections [8,418,462].

In addition to BSI, CoNS can also cause local infections when colonizing medical devices without access to the bloodstream. Drain-associated cases of meningitis/ventriculits [463], endophtalmitis [464,465], peritonitis [466] and cerebrospinal fluid shunt- [467], prosthetic joint- [468,469], mammary implants-[470,471], and surgical sites-associated infections [455,472] are some examples.

While more representative, CoNS are not only linked with the development of infections related to the use of IMD. Cases such as healthcare-associated native valve endocarditis in adults [473], meningitis [474], and necrotizing fasciitis [475] in preterm infants were also reported. Moreover, confirming CoNS versatility, several CoNS species have been also implicated in laryngological diseases (rhinosinusitis, sinusitis) and infections (frontal sinus, throat, larynx, nares, tonsils, and trachea infections) (reviewed in [476]).

### 7.2. Evasion from the Host Immune System

Amongst the strategies used by CoNS to protect themselves from the host immune system, biofilm formation is one of the most important [477]. This is partially related to the fact that biofilms are composed of molecules with important protective roles and because it harbors cells with a wide range of metabolic activities.

Regarding biofilm-associated molecules, PNAG, the major component of *S. epidermidis* biofilms, has a significant function in bacterial cell protection. PNAG was found to protect *S. epidermidis* biofilm cells from several host defense mechanisms such as neutrophils and macrophage killing, complement deposition, immunoglobulins, and antimicrobial peptides action (AMPs) [33,478,479,480]. It is noteworthy that PNAG deacetylation was shown to be crucial for immune evasion [481,482,483,484]. The mechanism by which PNAG protects cells from AMPs is often related to electrostatic repulsion (positive/positive charge). Interestingly, PNAG also protects against negatively charged AMPs, namely dermicin, suggesting that PNAG functions as a decoy by sequestrating oppositely charged AMPs [479]. Additionally, *S. epidermidis* has an AMP-sensing system that activates mechanisms that decrease the overall negative charge of the bacterial cell wall, thereby hindering the efficient attraction of cationic AMPs [485]. These include the D-alanylation of TAs [61], as described earlier, and the lysylation of membrane phospholipids by the MprF (also known as FmtC) [486]. The production of the extracellular enzyme SepA has an important function in the protection against AMPs, as it promotes AMPs’ proteolytic breakdown. Furthermore, it was reported that SepA confers protection of *S. epidermidis* cells against being killed by neutrophils [487]. There are, yet, other findings showing the pro-inflammatory effect of PNAG [478,488,489]. On the one hand, these apparently contradictory results may be related to the distinct models used in the different studies performed and due to the difficulty to attribute the observed effects directly to PNAG molecule as (i) it is challenging to obtain PNAG molecule in high purity and (ii) because PNAG-deficient strains have distinct cell surface properties, which together may influence the host response [490]. On the other hand, these results show the complex balance of the immune response elicited by *S. epidermidis*.

In addition to PNAG, *S. epidermidis* produces another exopolymer, the poly-γ-glutamic acid, whose production was found to be upregulated in the biofilm phenotype [491], and that has also been implicated in the defense against the host immune system attack. Although its primary function is to allow *S. epidermidis* to survive in high salt concentrations environments, like the human skin, it seems important to resist phagocytosis by neutrophils and AMP action [492]. Of note, the proteins Aap and Embp are involved as well in the protection of *S. epidermidis* biofilm cells as both seem to hamper macrophage phagocytosis [74,493].

PSMs also have a part in the protection of *S. epidermidis* cells against the investiture of the effectors of the host immune system. Among the PSMs produced, some present potent cytolytic activity against human neutrophils such as the PSMε and PSMδ [487,494]. Interestingly, although *S. epidermidis* has the potential to produce effective cytotoxins, these are produced in lower quantities [487]. These findings indicate that *S. epidermidis* prefers to employ a rather passive strategy to stimulate a low inflammatory profile and, this way, achieve a successful evasion from the host immune system [487].

Lastly, in regard to the metabolic heterogeneity of *S. epidermidis* biofilm cells and the advantage in the evasion from the host immune system, the presence of VBNC cells is an important factor (for more details, see Section 2). Earlier, it was demonstrated that *S. epidermidis* biofilms with higher proportions of VBNC are less inflammatory inducing, thus, less phagocytosis by murine macrophages, both in vitro and in vivo [172].

### 7.3. Diagnostics

As part of human flora, the diagnostic of the infections caused by CoNS is often confusing, as their presence in clinical samples does not unequivocally indicate infection, possibly being the result of contamination during sample collection [495]. Hence, it is puzzling to assess the clinical relevance of a positive culture frequently resulting in (i) significantly longer hospital stays, extra diagnostics, and treatments, (ii) application of unnecessary treatments that greatly contribute to the antibiotic selection pressure and, finally, (iii) delayed application of the adequate treatment regimen that ultimately can lead to patient mortality [8]. Consequently, to alleviate such issues, over the years, several studies have been performed in the direction of finding markers with the capacity to distinguish between *S. epidermidis* that live on the skin from those that cause infections. However, since the CoNS virulence factors are the same that confer its fitness as a commensal (reviewed in [42]), this task is rather challenging.

Based on several studies, it was observed that commensal strains seem to be more susceptible to antibiotics [496], often positive to the genes *aap* and *fdh* and the ACME element [154,496,497,498] and repeatedly negative to the biofilm-associated genes *icaA* and *bhp* and the IS256 [499,500]. In the case of clinical isolates, it has been often linked to higher antibiotic resistance and the presence of the genes *icaA* and *bhp*, as well as the carriage of the IS256 and SCC*mec* elements [496,497,498,501,502,503]. In addition, a phylogenetic analysis of *S. epidermidis* isolates from healthy human skin infections showed the presence of two separate clusters, the lineage A/C and B [154,504]. The strains belonging to the first lineage contained most of the isolates from colonization and infection, while the lineage B was mainly composed of colonization isolates [154,504]. Recently, the genotypic and phenotypic differences between both lineages were characterized supporting the higher pathogenic potential of the strains belonging to the A/C lineage and the potential of *fdh* to be used as a marker for commensal isolates [505].

Even so, a novel diagnostic strategy based on these putative markers is not yet under use or, as far as we know, under consideration. As such, more studies are necessary. So far, the majority of the studies have relied on DNA or phenotype analysis; however, considering that gene transcription is altered depending on the conditions of the involving environment, the analysis on how commensal and clinical isolates respond in the course of infection by analyzing its transcriptome may hold the key to find suitable markers.

### 7.4. Alternative Treatment Strategies

Aside from the difficulty associated with the accurate diagnosis of CoNS infections, the increasing resistance of CoNS to multiple antibiotics agents together with the high tolerance to antibiotics demonstrated when growing in biofilms [31], are critically reducing the treatment options since antibiotics remain the primary form of treatment. Thus, a serious effort has to be made to manage the plethora of infections caused by CoNS as these primarily affect a growing and susceptible population of our society. As such, in the last few decades, researchers have been tackling this issue from different angles.

#### 7.4.1. Immunoprophylaxis and Immunotherapy Strategies

Considering that the majority of the infections caused by CoNS are associated with biofilm formation on IMD, surface and matrix molecules involved in this process were initially addressed [506,507,508]. PNAG was one of the first molecules to be targeted due to its role in immune evasion and also because it is the principal mediator of biofilm formation in staphylococcal species. In *S. epidermidis*, it was shown that human monoclonal antibodies (mAbs) against PNAG were effective in killing planktonic and biofilm cells in opsonophagocytic in vitro assays [33]. In addition, it was shown that mAbs anti-PNAG inhibited biofilm accumulation in vitro and were protective in a rabbit endocarditis model [509]. However, biofilm accumulation inhibition in the presence of mAb anti-PNAG seems to be strain-dependent [510]. The potential of several proteins such as Aap, SesC, and SdrG as target candidates for antibody-based therapies was also considered. Biofilm formation by *S. epidermidis* was impaired by mAbs anti-Aap, but in a biomaterial-associated infection model, neither enhanced opsonophagocytosis nor protected mice were observed [511,512]. On the other hand, polyclonal rabbit sera against SesC were shown to significantly inhibit *S. epidermidis* biofilm formation in vitro and in vivo and vaccination with recombinant SesC reduced *S. epidermidis* biofilm formation and infection rate in an animal model [73,103]. The incubation of *S. epidermidis* with antibodies anti-SdrG previous to challenge reduced the bacterial load in the kidneys of infected mice [513]. Later on, it was shown that previous vaccination of mice with antibodies anti-SdrG conferred protection [514]. Due to their relevant role in biofilm formation and maintenance, TAs [515,516] and β-type PSM [114] were also explored. Nevertheless, despite encouraging results, anti-staphylococcal vaccines or immunotherapy strategies have failed clinical trials [517,518,519]. By analyzing all failed attempts, it became clear that targeting a single antigen has limited success and, thus, a multivalent approach would increase the chances of developing effective vaccines. Hence, new advances were made in the past few years, reviving the interest in developing vaccines against infections caused by staphylococcal species [507,520].

#### 7.4.2. Interfering Molecules

To prevent or eradicate staphylococcal biofilms, a vast range of substances with different mechanisms have been identified (reviewed in [129,521]. As mentioned before, dispersin B degrades PNAG, leading to biofilm dispersion [119]. In addition, DNases and proteases, which may be self-produced, can be used to interfere with the stability of eDNA- and protein-based biofilms, respectively. Nevertheless, the application of dispersing agents has limitations as it leads to the spreading of bacterial cells and may also result in an aggressive response of the host immune system [127]. As such, it was established that dispersion agents need to be applied together with antibiotics to be effective. One shall consider that the cells released from biofilms have a particular phenotype showing, in *S. epidermidis*, higher tolerance to a few antibiotics [126]. In addition, it is important to consider the side effects of applying molecules with broad activity, such as proteases, as these may interfere with host proteins and tissues [129]. Other interesting molecules already discussed in this review are bacteriocins. Gallidermin [435] and nisin [522], for instance, seem to be able to efficiently prevent *S. epidermidis* biofilm formation and disrupt established biofilms, respectively. Finally, the use of molecules with the capacity to arrest QS communication among cells, called quorum quenching molecules, is being revisited and promising results were obtained in *S. aureus* [523].

#### 7.4.3. Phages and Phage-Derived Enzymes

Another interesting strategy to treat biofilm-related infections is the use of phages and phage-derived enzymes (reviewed in [524,525]) as these present a narrow host specificity preventing, thus, the killing of beneficial bacteria during treatment. In addition, phages can affect antibiotic-susceptible and -resistant bacteria [526].

Phages can be used alone or in combination with other molecules such as antibiotics or dispersion agents and, to increase the efficacy of the treatment, two or more phages can be mixed [129]. Phage K is a well-documented polyvalent staphylococcal phage with reported activity against *S. epidermidis* biofilm cells [527]. More recently, a *S. epidermidis*-specific phage (SEP1) was shown to infect different *S. epidermidis* planktonic cells, namely on exponential and stationary phases [528]. Although not able to infect intact biofilms, SEP1 was able to infect scraped biofilms, persister and biofilm-released cells, suggesting that its activity was affected by the biofilm matrix [85].

Regarding phage-derived enzymes, the endolysin LysGH15 was able to eliminate planktonic cells, as well as to inhibit and disrupt biofilms formed by *S. epidermidis*, *S. haemolyticus*, and *S. hominis*. Moreover, the efficacy of LysGH15 was analyzed in vivo and a lower bacterial load was observed in the blood and solid organs when compared with the control [529]. Another lysin, the CF-301, has also shown to be efficient against biofilms formed by several CoNS species, on different surfaces, including mixed-species composed of *S. aureus* and *S. epidermidis* [530]. In another study, a phage-origin extracellular polymeric substance (EPS)-depolymerase (Dpo7) was shown to be able to prevent and disperse staphylococcal biofilms in polysaccharide-dependent biofilm forming strains [531].

Notwithstanding, despite the efforts made, the current existing strategies to fight staphylococcal infections consist of antibiotics and preventing the colonization of medical devices before implantation by increasing hygiene and disinfection measures [477].

## 8. Enterotoxins and Impact on Food

While the highly virulent *S. aureus* is usually responsible for acute infections, CoNS mostly differs from *S. aureus* by being less virulent, being frequently associated with chronic infections. Generally, CoNS pathogenicity is associated with some molecular mechanisms that evolved for a commensal lifestyle on the skin that can have extra use throughout infection development [42]. In opposition to *S. aureus*, generally, CoNS strains do not produce aggressive toxins [42]. Staphylococcal enterotoxins compose a family of toxins that are analogous both chemically and biologically. Upon ingestion of these toxins, a disease called staphylococcal food poisoning (SFP) can occur. This usually happens due to improper handling or storage of staphylococcal contaminated foodstuff, such as meats, salads, creams, and dairy products. In 2012, the CDC estimated that, in the United States of America, SFP caused >240,000 illnesses leading to >1000 hospitalizations and six deaths per year [532]. This happens usually on meats, salads, creams (bakery), and dairy products [533]. The presence of *S. aureus* in food is considered a public health hazard for its ability to produce enterotoxin and the risk of development of consequent food poisoning. After ingestion of the toxin, a typical incubation period of 6–10 h is expected. Usual symptoms include headache, nausea, abdominal cramps, vomiting, general weakness and prostration, dizziness and chills, and diarrhea (sometimes containing blood) [534]. To date, there are nine different staphylococcal enterotoxins identified that are designated as A, B, C1, C2, C3, D, E, F, and G. However, enterotoxins A and D are responsible for the majority of the outbreaks [535]. These toxins are members of the pyrogenic toxins family that have the capability to stimulate a high percentage of T cells, thereby acting as superantigens. They are highly thermostable and therefore difficult to inactivate in the human body [534]. In a recent study with more than 1500 staphylococcal genomes analyzed, enterotoxin-encoding genes were detected in 97% of the *S. aureus* genomes (857 out of 883), while only nine genes were detected in *S. epidermidis*. Around 70% of the *S. aureus* genomes were reported to encode genes forming enterotoxin gene clusters, where the *selx* gene was found to be the most frequent (782 strains). The nine *S. epidermidis* strains mentioned encoded both *sec* and *sel* genes. In the mentioned study, a phylogenetic analysis was also performed, and the authors observed that all nine enterotoxigenic *S. epidermidis* strains belonged to a cluster of 65 strains very distant from the other 499 strains [374]. Despite these rare reports about the presence of enterotoxins in CoNS, their impact on virulence is still not clear [358]. CoNS strains have rarely been associated with food poisoning as they usually do not grow rapidly in foods. However, some enterotoxin-producing CoNS strains have been isolated from cases of SFP [536,537]. However, although with low frequency, several studies reported that different CoNS isolated from poultry can encode toxin-producing genes [538,539]. CoNS species such as *S. epidermidis*, *S. gallinarum*, *S. arlettae*, *S. chromogenes*, and *S. xylosus* have commonly been isolated from the skin and nares of chickens [540]. Despite being part of chicken microbiota as harmless colonizers, it is now accepted that some of these species can be pathogenic under specific conditions. Indeed, commensal strains are seen as a reservoir of antibiotic-resistant genes, and that justifies why slaughter poultry has been recognized as one of the most important vehicles for the dissemination of antimicrobial resistance genes [21]. CoNS are stated as playing a major role in the development of sensory properties in fermented foods and sausages [541]. Particularly, *S. carnosus*, *S. equorum*, *S. succinus*, and *S. xylosus* are known to produce low molecular-weight compounds that have a high impact on product flavor [541]. Moreover, for safety reasons, several CoNS strains have been selected as starter cultures in meat fermentation processes [542].

As abovementioned, some CoNS, namely *S. epidermidis*, *S. haemolyticus*, and *S. saprophyticus*, have been associated with nosocomial infections, and these species are also frequently associated with foodstuff. Genomic analysis of these strains proved that CoNS are a reservoir of antibiotic-resistant genes. As *S. aureus* and *S. epidermidis* usually inhabit similar ecological niches, gene flow between these two species is predicted to occur with high prevalence. This can ultimately lead to the emergence of *S. epidermidis* toxigenic strains. Consequently, in the future, the possibility of SFP caused by CoNS strains should be considered.

## 9. Conclusions

As clearly shown in this review series, CoNS are a versatile group of staphylococcal species that are equipped with the necessary factors and strategies to withstand the host and/or involving environment stresses. In regard to CoNS clinical implications, considering that modern medicine mainly relies on the use of medical devices and the current shift in patient’s demographics towards increased numbers of vulnerable patients, it is likely that CoNS-caused infections will become even more frequent, contributing to overall morbidity, mortality, and socioeconomic distress. When it comes to CoNS repercussion in the food processing environment, while the production of enterotoxins has been described in CoNS, these are not generally accepted as enterotoxins producers and, as such, the role of CoNS in foodborne diseases has been overlooked. However, because CoNS are commonly found in food and share the same niche with *S. aureus*, through HGT events, it is predicted that the interest in these species as foodborne pathogens will increase over the next years. However, much remains to be done to further comprehend the involvement of CoNS in the emergence of foodborne diseases.

Overall, considering that antibiotics are still our primary form of treatment and that these are greatly inefficient against CoNS-caused infections, in particular against biofilm-originated infections, there is an urgent need to find new alternatives. To overcome this challenge, it is necessary to endorse more basic and clinical research aiming to (i) underpin CoNS colonization mechanisms, reservoir function, and the dichotomy commensal/pathogen; (ii) elucidate the mechanisms promoting antibiotic resistance, as well as tolerance; (iii) characterize CoNS in-host evolution as well as the host response. In addition, further applied research addressing the development of alternative methods to prevent and/or eradicate CoNS biofilms such as (i) the development of new biomaterials and coatings to avoid bacteria initial attachment, (ii) the search for effective phages and phage-derived enzymes as well as (iii) interfering molecules need to be supported. Only this way will the scientific community have the means to seriously tackle this issue and, at last, develop effective strategies to control the infections caused by CoNS.

## Figures and Tables

**Table 1:** Reports of antimicrobial resistance of the 20 more frequently isolated CoNS against the 20 main antibiotics used in clinical and veterinary settings.

		***S. capitis*** ***S. urealyticus***	***S. caprae***	***S. carnosus*** ***S. utilis***	***S. cohnii*** ***S. urealyticus***	***S. condimenti***	***S. epidermidis***	***S. equorum*** ***S. linens***	***S. haemolyticus***	***S. hominis*** ***S. novobiosepticus***	***S. lentus***
Cell wall synthesis inhibitorsn	Ampicillin	+[280]	+[281]	+[282]	+[280]	+[283]	+[284]	+[280]	+[284]	+[280]	+[283]
	Cefazolin	+[280]	+[280]	NF ^2^	+[280]	NF ^2^	+[191]	+[280]	+[280]	+[280]	+[285]
	Fosfomycin	+[257]	+[286]	NF ^2^	+[287]	+[288]	+[289]	NF ^2^	+[290]	+[287]	+[287]
	Imipenem	+[280]	+[291]	NF ^2^	+[280]	NF ^2^	+[291]	NF ^2^	+[280]	+[280]	NF ^2^
	Methicillin	+[256]	+[281]	+[292]	+[293]	NF ^2^	+[294]	+[293]	+[294]	+[294]	+[294]
	Penicillin	+[295]	+[296]	+[297]	+[284]	+[283]	+[284]	+[298]	+[284]	+[299]	+[300]
	Oxacillin	+[301]	+[296]	+[282]	+[296]	NF ^2^	+[284]	+[302]	+[299]	+[299]	+[300]
	Teicoplanin	+[303]	+[304]	NF ^2^	+[305]	NF ^2^	+[298]	+[302]	+[290]	+[302]	+[306]
	Vancomycin	+[256]	+[307]	+[297]	+[308]	NF ^2^	+[303]	+[302]	+[304]	+[304]	+[309]
NA ^1^ synthesis inhibitors	Ciprofloxacin	+[310]	+[296]	+[297]	+[296]	NF ^2^	+[310]	+[302]	+[310]	+[300]	+[300]
	Levofloxacin	+[301]	NF ^2^	+[311]	+[191]	NF ^2^	+[300]	NF ^2^	+[301]	+[301]	+[300]
	Rifampicin	+[256]	+[286]	NF ^2^	+[312]	+[313]	+[302]	+[282]	+[284]	+[290]	+[22]
Protein synthesis inhibitors	Clindamycin	+[301]	+[286]	+[311]	+[284]	NF ^2^	+[284]	+[302]	+[284]	+[301]	+[300]
	Erythromycin	+[301]	+[296]	+[297]	+[284]	+[313]	+[299]	+[284]	+[299]	+[299]	+[314]
	Gentamicin	+[301]	+[296]	+[297]	+[296]	NF ^2^	+[310]	+[315]	+[299]	+[299]	+[300]
	Linezolid	+[295]	NF ^2^	+[316]	+[317]	NF ^2^	+[318]	+[302]	+[300]	+[300]	+[300]
	Quinupristin- Dalfopristin	+[319]	+[311]	NF ^2^	+[284]	NF ^2^	+[284]	NF ^2^	+[284]	+[320]	+[321]
	Tetracycline	+[284]	+[322]	+[297]	+[284]	+[283]	+[284]	+[284]	+[299]	+[299]	+[300]
	Tigecycline	NF ^2^	+[311]	+[311]	+[311]	NF ^2^	+[284]	NF ^2^	+[323]	+[300]	+[300]
Alter. cell membrane	Daptomycin	+[301]	NF ^2^	NF ^2^	+[311]	NF ^2^	+[303]	NF ^2^	+[324]	+[324]	+[283]
		***S. lugdunensis***	***S. pasteuri***	***S. piscifermentans***	***S. saprophyticus*** ***S. bovis***	***S. sciuri***	***S. vitulinus***	***S. simulans***	***S. succinus*** ***S. casei***	***S. warneri***	***S. xylosus***
Cell wall synthesis inhibitors	Ampicillin	+[280]	+[218]	NF ^2^	+[280]	+[280]	+[315]	+[280]	+[282]	+[284]	+[284]
	Cefazolin	+[280]	NF ^2^	NF ^2^	+[280]	+[280]	NF ^2^	+[280]	NF ^2^	+[280]	+[280]
	Fosfomycin	+[325]	NF ^2^	+[326]	+[327]	+[220]	NF ^2^	NF ^2^	NF ^2^	+[289]	+[306]
	Imipenem	NF ^2^	NF ^2^	NF ^2^	NF ^2^	NF ^2^	NF ^2^	NF ^2^	NF ^2^	+[328]	NF ^2^
	Methicillin	+[293]	+[329]	+[330]	+[294]	+[293]	+[331]	+[294]	+[332]	+[294]	+[293]
	Penicillin	+[295]	+[284]	+[330]	+[284]	+[284]	+[296]	+[295]	+[333]	+[284]	+[284]
	Oxacillin	+[296]	+[299]	+[330]	+[300]	+[300]	+[299]	+[295]	+[282]	+[299]	+[299]
	Teicoplanin	+[334]	NF ^2^	NF ^2^	+[305]	+[319]	NF	+[319]	NF ^2^	+[304]	+[335]
	Vancomycin	+[24]	NF ^2^	+[330]	+[330]	+[330]	+[307]	+[336]	+[316]	+[304]	+[330]
NA ^1^ synthesis inhibitors	Ciprofloxacin	+[296]	NF ^2^	NF ^2^	+[300]	+[300]	+[296]	+[295]	NF ^2^	+[300]	+[310]
	Levofloxacin	+[334]	NF ^2^	NF ^2^	+[300]	+[300]	NF ^2^	+[320]	NF ^2^	+[300]	+[311]
	Rifampicin	+[337]	NF ^2^	+[330]	+[338]	+[220]	+[315]	+[22]	+[316]	+[301]	+[22]
Protein synthesis inhibitors	Clindamycin	+[295]	+[270]	+[330]	+[300]	+[284]	+[333]	+[295]	+[270]	+[284]	+[284]
	Erythromycin	+[296]	+[299]	+[312]	+[300]	+[300]	+[299]	+[284]	+[270]	+[299]	+[299]
	Gentamicin	+[296]	+[299]	NF ^2^	+[300]	+[191]	+[299]	+[295]	+[316]	+[299]	+[299]
	Linezolid	+[339]	NF ^2^	NF ^2^	+[330]	+[300]	+[340]	+[295]	NF ^2^	+[295]	+[300]
	Quinupristin- dalfopristin	NF ^2^	NF ^2^	NF ^2^	+[319]	+[284]	NF ^2^	+[284]	NF ^2^	+[320]	+[311]
	Tetracycline	+[334]	+[299]	+[282]	+[284]	+[333]	+[299]	+[284]	+[314]	+[299]	+[299]
	Tigecycline	+[323]	NF ^2^	NF ^2^	+[300]	NF ^2^	NF ^2^	+[284]	NF ^2^	+[323]	+[22]
Alter. cell membrane	Daptomycin	NF ^2^	NF ^2^	NF ^2^	+[283]	+[284]	NF ^2^	NF ^2^	NF ^2^	+[301]	+[283]

^1^ NA, nucleic acids; ^2^ NF—Not found.

## Data Availability

No new data were created or analyzed in this study. Data sharing is not applicable to this article.
